# Difficulties and Perspectives of Immunomodulatory Therapy with Mistletoe Lectins and Standardized Mistletoe Extracts in Evidence-Based Medicine

**DOI:** 10.1093/ecam/nep191

**Published:** 2011-01-09

**Authors:** Tibor Hajtó, Krisztina Fodor, Pál Perjési, Pèter Németh

**Affiliations:** ^1^Department of Immunology and Biotechnology, University of Pécs, Pécs, Hungary; ^2^Department of Medical Chemistry, University of Pécs, Pécs, Hungary

## Abstract

*Viscum album* preparations are aqueous mistletoe plant extracts used in complementary and alternative medicine as immunomodulators in cancer therapy. However, evidence of immunological efficacy of mistletoe extracts (MEs) used in clinical trials is often lacking. Mechanisms involved in anti-tumor properties of ME and mistletoe lectins (MLs) modify both innate and adaptive immune systems, according to animal model experiments. In the background of these effects, a selective binding of ML on CD75 ganglioside receptors of interleukin 12 (IL-12)-producing macrophages or dendritic cells can play an important role. Immunological effects of ME correlate with their lectin activity, showing a bell-shaped dose-response curve of efficacy. Therefore, a correct determination of MLs for the standardization of commercial ME is essential. However, plant MLs exhibit heterogeneity, which most likely results from post-translational processing. In addition, amino acid analysis of ML has revealed numerous conservative substitutions along their amino acid sequence. Consequently, ML research needs new perspectives, and the advantages and disadvantages of purified and biologically better defined ML preparations are also discussed in this article.

## 1. Introduction

*Viscum album* (VA) is a parasitic plant that grows on various trees. It is commonly known as European mistletoe. VA preparations are aqueous extracts used as a complementary medicine in cancer therapy. Various clinical studies reveal that mistletoe extract (ME) preparations can improve the quality of life in different cancer patients. The most important biologically active components of ME preparations are mistletoe lectins (MLs). Other constituents, such as viscotoxins, Kuttan peptide, polysaccharides, alkaloids, viscin and vesicles, have also been investigated; but their exact *in vivo* role in biological effects of whole ME are still unclear. Treatment with lectin-containing ME preparations or purified ML is associated with tumor regression in several *in vivo* experimental models. Mechanisms by which ME affect cancer cells have been described for various cellular activities, namely apoptosis [1–5], cell cycle [6, 7], protein synthesis [8], angiogenesis [9] and immunomodulation [9–13]. Experiments in animal models suggest that ME- and ML-mediated inhibition of tumor growth is associated with their immunomodulatory efficacy. The mechanisms involved in anti-tumor properties include an enhancement of interleukin 12 (IL-12) secretion and natural killer (NK) cell function, which point to an improved balance of the innate immune system [14]. Case reports and preliminary clinical observations support these experimental results [15, 16]. Consequently, this review summarizes difficulties in the immunologically effective and reproducible application of ML and standardized ME in clinical trials. Two essential problems will be discussed here: (i) the lack of immunological concepts and evidence in clinical trials carried out with various ME and (ii) difficulties and perspectives with a standardized and reproducible application of ML and ME.

## 2. Difficulties of the Evidence-Based Judgment of Immunotherapy by MEs and MLs

### 2.1. Successful Immunotherapy against Cancer Requires New Clinical Concepts

**Figure 1:**
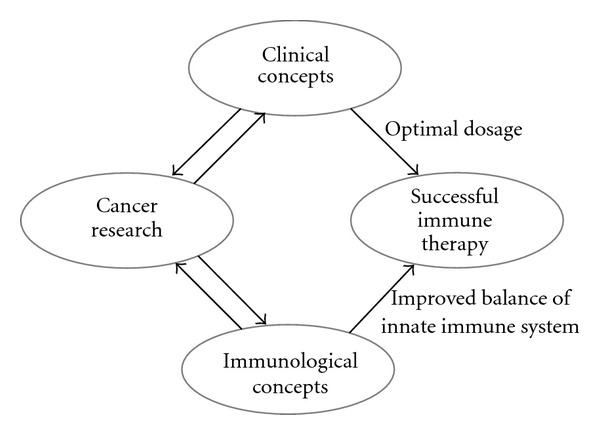
Schematic illustration of the necessity of relationships between cancer research and immunological and clinical concepts for a successful immune therapy.

In spite of substantial experimental data, the clinical relevance of the immune system in tumor disease is often insufficiently understood, and the correct judgment of the rather complex immune system in tumor defense is often controversial.  Figure 1 tries to give a schematic illustration about this problem. Clearly, not all immune mechanisms are impaired during tumor disease, but the decrease of several functions of innate immune system supports the hypothesis that they may contribute to the tumor's escape from immune destruction. However, the usual clinical and laboratory investigations of tumor patients are often unable to detect any signs of an immune deficiency because the most highly developed specific immune functions of patients during tumor progression can remain within normal range. Despite this discrepancy, experimental research has regularly found growing evidence that depression of numerous innate immune functions correlates with progression of cancer. For example, when the interaction between specific immune responses and non-specific inflammatory reactions and their relation with prognosis of cancer patients were analyzed, results revealed that although there was a significant specific anti-tumor response as reflected by T cells, their effects on patient survival and local recurrence were less important when compared with effects of non-specific inflammatory responses [17]. In addition, defects in the major histocompatibility complex class I antigen have been described in tumors of different histopathology, which can hinder the effectiveness of T lymphocytes [18]. 

Numerous experiments have attempted to find reasons for the decreased activity of the innate immune system in tumor patients, and there is agreement that soluble factors produced or induced by malignant cells play an important role in this depression [19]. In addition, myeloid-derived suppressor cells have also been found to be responsible for this phenomenon [20]. In spite of the fact that basic functions of the innate immune system are depressed in tumor patients, its investigation is not in current clinical praxis, causing a continuous lack of fresh clinical experience and emerging concepts. This lack of understanding in evidence-based medicine has hindered development of various kinds of non-specific immunotherapy modalities against cancer. Also, a periodic assessment of the suppressive nature of the tumor microenvironment would also be helpful, although not practical, to better understand these unspecific immunotherapeutic interventions.

**Figure 2:**
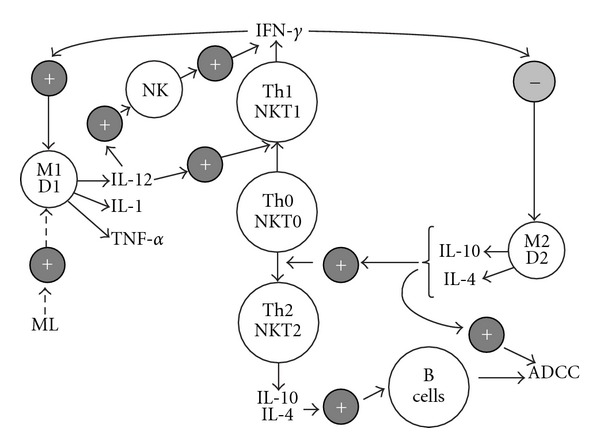
Balance of innate immune system (schematic and simple represented by arrows with directed lines) and presumable effect of ML *given with ME* (represented by arrow with broken lines). The signal “+" means a stimulatory effect and “−" indicates an inhibitory effect. As shown, ML stimulates pro-inflammatory cytokines and IL-12-producing macrophages and dendritic cells [11, 12, 23]. This figure represents a modified version of an illustration published by Murray [24]. ADCC: antibody-dependent cell-mediated cytotoxicity.

We must not forget that inflammation can exhibit controversial effects. It may eradicate tumor cells but, when chronic, may also promote tumor growth. As shown in Figure 2, M1 macrophages and DC1 dentritic cells generate IL-12, pro-inflammatory cytokines and activate cytotoxic effector cells, such as NK and NKT cells, which are potent inhibitors of tumor growth. However, they are defective in tumor patients. Available information suggests that tumor-associated macrophages belong to a prototypic M2 population [21]. M2 generates IL-4 and IL-10, which facilitate the generation of T helper 2 (Th2) cells and inhibit Th1 cells [22]. M2 macrophages affect inflammation and promote cell proliferation by producing growth factors and products of the arginase pathway as well as promoting angiogenesis and tissue repair [21]. 

Tumor patients can have up to 40% more M2 peripheral monocytes than healthy individuals, who have only 10% M2 monocytes [22]. NKT cells can also have a similar opposing effect. In cancer, NKT-1 cells are protective, producing interferon-*γ* (IFN-*γ*) to activate NK and DC1 dendritic cells that produce IL-12. NKT-2 cells primarily inhibit tumor immunity [25]. These findings indicate an impaired balance of the innate immune system in cancer patients. Consequently, learning to manipulate this balance along the regulatory axis may be critical to devise successful immune therapies against cancer.

### 2.2. Treatment with ME, as One of the Most Widely Used Alternative Immunomodulatory Treatment of Cancer Patients in Europe, Is Often Not Related to Clear Clinical Immunological Concepts

In the last decade, clinical study of complementary immune therapy using various plant extracts has progressed slowly, and the lack of clear immunological concepts often contributes to this negligence.  Figure 1 shows a simplified illustration of the relationships between cancer research, immunological concepts and clinical concepts. Clinicians often have a feeling that too much is demanded of them. In the literature of complementary medicine concerning tumor immunology, there are often speculative *pro* and *contra* arguments. Consequently, it is also not surprising that in a great number of clinical trials the doses of ME are not reported; such reports would have enabled a more exact and reproducible chemical and immunological definition for using this therapy. Moreover, in spite of emphasis on the generally accepted opinion that both cytotoxic/apoptosis-inducing and immunomodulatory effects are important in the clinical benefit of ME, the immunological results are lacking in most clinical reports published in the last 20 years. The latter would have been able to strongly support the beneficial immunological effectiveness of ME preparations. This deficiency is therefore surprising, because many years ago a bell-shaped dose-response relationship of ME-induced immunological effect was established [9–13]. This indicated that optimal doses are necessary for clinical trials.

## 3. Clinical and Immunological Attempts for Monitoring the ME-/ML-Induced Improvement of Immune Balance in Tumor Patients

### 3.1. Why Is the Investigation of NK Cells Emphasized?

As mentioned above, tumor immunity seems to be restricted to the M1/D1/NKT-1 pathway of innate immunity, and therefore these natural immune mechanisms must be taken into consideration for successful immunotherapy against cancer [26]. Activation of this pathway by ML enhances cytotoxic functions of NK, *γδ*T and NKT cells, which also produce IFN-*γ*, and further stimulate M1/D1 cells and inhibit M2/D2 cells (Figure 2). Consequently, the immunological research of ME and ML focused on the NK system, which seems to be available for monitoring the M1/D1/NKT-1 pathway of the innate immune system [11–13].

In previous studies, investigations of NK cells allowed active dose-dependent results with ML and standardized ME. In addition, NK cells were also stimulated by ML-I *in vitro,* which was, in an additive manner, enhanced by its combination with IL-2 and IL-12 [11, 12]. *In vivo*, ML-I stimulates the activity and peripheral levels of NK cells showing a bell-shaped curve of efficacy. Studies on animal models show that application of 0.5–3 ng/kg ML-I twice a week is effective to sustain elevation in the number and activity of peripheral blood NK cells. If lectin injections were given more frequently (daily), the NK system was not stimulated, indicating that frequent application may lead to a situation similar to that regularly observed in chronic inflammation with dominance of the M2/D2/NKT pathway [11, 12]. Moreover, in healthy persons there is often a high intrinsic fluctuation in NK activity and frequency. Blind crossover studies have revealed an optimal lectin dose of *∼*0.5 and 1 ng/kg if given twice week [27]. These results suggest the potential use of ML and standardized ME as modulators that can manipulate the balance of the innate immune system in a clinically more successful direction.

However, most clinical trials using ME do not take into consideration results of immunological research. Non-optimal and higher doses of ML and ME do not induce any significant responses in the innate immune system. It must not be forgotten that cytotoxic effects of ME can down-regulate their immunological effects if they are not given in optimal dose [11]. Consequently, the lack of immunological evidence in many clinical trials with ME makes an objective judgment of its immunomodulatory potential difficult.

### 3.2. New Perspectives for Immunotherapy with ML and Standardized ME

It is well known that novel immunotherapeutic approaches such as DNA vaccines, dendritic cell preparations, heat shock protein-based vaccines and gene transfer technology demonstrated exciting results in animal experiments, although their evaluation in clinical trials showed no exceptional tumor protection in a significant number of patients [28]. Consequently, growing evidence suggests that the effectiveness of tumor-specific adaptive immune responses induced by various vaccinating agents can be enhanced by parallel activation of the appropriate component of the innate immune system [28].

The concept of cancer immunotherapy with ML provides fresh perspectives as it may avoid many of the drawbacks of conventional therapies, such as chemotherapy, irradiation and surgery. Targeting the innate immune system in cancer is of growing importance [28]. Conventional therapy modalities alone do not improve the impaired immune balance of tumor patients. For example, if the impaired immune balance of tumor patients before and after chemotherapy is compared, independent of clinical responses, no differences are observed [29].

## 4. Pharmacochemical Difficulties in Standardization and Reproducible Application of ME and ML Preparations

### 4.1. Which Components of ME Are Important in Their Immunological Standardization?

As already mentioned, not only lectins but also other components such as viscotoxins [30], Kuttan peptide [31], polysaccharides [32] and vesicles [33] have been suggested by several authors for participating in the immunomodulatory efficacy of ME. However, up to now, these mistletoe components have only been tested *in vitro*; only MLs have been verified *in vivo* as substances responsible for the immunological effects of ME [10, 12, 13, 27]. In a previous study, all types of ML were completely removed from a commercially available ME preparation by chromatographic procedures without causing any other further alteration in the composition of the extract. The removal of ML from the immunologically effective ME resulted in immunosuppressive responses in healthy volunteers injected with the lectin-free preparation [10]. This residual immunotoxicity of lectin-free extracts may be related to viscotoxins, which can cytolytically damage cell membranes [34]; other components, such as viscin [35] may also be involved. Consequently, for the immunological standardization of MEs, the determination of active MLs is essential.

### 4.2. Difficulties with Lectin Standardization of ME and ML Preparations

For the immunological research of ML and ME, a standardization procedure, namely the enzyme-linked lectin assay (ELLA), was modified [10] and optimized [36] so that the binding capacity of MLs from plants and extracts to asialofetuin is measured. Since the method is based on binding lectin to an immobilized oligosaccharide ligand, the results of the ELLA assay showed a correlation with the lectin-induced immunological responses observed in *in vivo* experiments [10, 12, 13, 27].

As already mentioned, in the standardization of commercial ME, the correct determination of MLs plays an important role. However, plant MLs exhibit a heterogeneity that most likely results from the post-translational processing of ML-I to the isoforms ML-II and ML-III [37]. Only a small difference was found in their primary structures. The antigenic analysis of B-subunit in ML-I and ML-III showed one epitope ^25^RDDDFRDGNQ^34^ in ML-I that is absent in the B chain of ML-III, and this difference can be related to some gene polymorphism [38]. ME preparations vary with regard to the content of ML-isoforms, which also may depend on the method of isolation or on various degradation effects. The chemical definition of ML-II and ML-III is based on lower molecular weights, small differences in primary structures and observations that *N*-acetyl-d-galactosamine (GalNAc) exhibits a more marked inhibition on ML-II- or ML-III-induced hemagglutination or cytostatic activity than the galactose-specific ML-I. In a previous study, MLs were carefully isolated from fresh plants and commercial MEs by ultrafiltration and affinity chromatography. The direct binding capacity of ML to lactose and GalNAc was compared in the same system. Surprisingly, no direct binding to immobilized GalNAc was detected. Only immobilized lactose was able to bind ML from the specially prepared extract. These unexpected findings could be interesting for further research, which may require new perspectives to find the appropriate binding sites and ligands.

The complete amino acid sequences of the A- and B-chains of ML-I have been determined [39, 40]. The A-chain contains 254 amino acid residues, and using matrix-assisted laser desorption ionization mass spectrometry (MALDI-MS), the existence of a potential *N*-glycosylation site was confirmed [39]. The B-chain is composed of 264 amino acid residues, and three potential *N*-glycosylation sites were confirmed by MALDI-MS analysis [40]. In addition, the B-chain consists of six subdomains, but only two of them (1*α* and 3*γ*) have sugar-binding receptors, namely the key residues in 1*α* receptor, Asp^22^, Gln^35^, Trp^37^, Asp^46^ and Gln^47^, and in 3*γ* receptor, Asp^234^, Ile^246^, Tyr^248^, Asn^255^ and Gln^256^. Therefore, the B-chain is the lectin part of the whole molecule [40].

Many years ago it was established by 2D gel electrophoresis that there are at least 40 isolectins of ML [41]. For chemical standardization of the ME preparations applied clinically, the exact determination of isolectin patterns is difficult. Amino acid analysis revealed 17 conservative substitutions along the amino acid sequence of the A-chain [39]. Analyzed sequence data of B-chain also show 12 conservative substitutions, most of them located in the C-terminal region of the protein [40]. Because of the heterogeneity of ML isoforms, an exact immunological standardization of commercial ME is not easy.

### 4.3. Using Plant Extract versus Purified VAA Fragment

Previous studies with ME revealed that the immunomodulatory effects and sugar-binding activity of an extract have a close relationship [11]. Therefore, it would be advisable to employ a standard procedure for exact and reproducible determination of the sugar-binding potency of ML and ME preparations using immobilized ligands with higher affinity for ML than asialofetuin. Recently, a highly specific receptor, the CD75 gangliosides, was described [42, 43], which is found on numerous effector cells of the innate immune system [44]. The existence of CD75 receptors may explain the selective binding capacity of neutrophils and monocytes to ML [23]. Consequently, ML or their sugar-binding fragments may be important candidates for an immunotherapy with a clearly defined targeting strategy. As shown in Table 1, these purified ML preparations show several advantages compared with plant extracts, since the translation of ME-indeed *in vitro* and preclinical results into clinical response continues to pose a problem [45, 46]. However, patients taking complementary and alternative medicine (CAM) are often satisfied with ME treatment because of good subjective results affecting their general status and fatigue [47]. Commercially available MEs are mostly given subcutaneously with various frequencies as a complementary therapy along with traditional cancer treatment. However, an exact summary of the results obtained with ME in human cancer therapy is not possible because the application of ME is rather heterogeneous and in many cases not reproducible. In addition, without appropriate standardization, ME and ML may induce immunological side effects, as it was found after high lectin doses in several cellular immune parameters were tested [10, 12]. As mentioned in Table 1, MEs in high and non-optimal doses can induce more side effects because apart from the toxic effect of overdosed ML, other toxic substances (such as viscotoxins and viscin) can also be involved. Using standardized lectin preparations and fragments may act as a bridge between the pharmaceutical industry and CAM. Table 1 summarizes the advantages and disadvantages of plant extracts and purified VAA preparations. 

## 5. Conclusions and Future Perspectives

1. Successful immunotherapeutic interventions by ML and ME must be associated with a lectin-induced improved balance of innate immunity in the tumor microenvironment. An immunologically optimized and standardized application of ML and ME may be helpful in enhancing the quality of life and prolonging tumor-free survival.
2. Further pharmacochemical research is necessary to introduce appropriate standardization procedures that allow better reproducibility for mistletoe preparations.
3. MLs and their fragments may be important candidates for immunotherapy with targeting strategy.

## Figures and Tables

**Table 1:** Advantages and disadvantages of ME and purified (or synthetic) ML preparations for clinical use.

Plant ME	Purified or fragmented biologically active ML
Advantages	
Easy to produce	Chemically well defined
Inexpensive	Biological effect is reproducible
Now available in pharmacy	Dosage calculation is exact
More clinical experiences	Less possible side effect
	Easy to adapt to CAM therapy

Disadvantages	
Mixture of different	Expensive to produce
unknown molecules with	
different biological activity	
Biological effect is less	Not available in pharmacy
reproducible	
Dosage calculation is difficult	Lack of clinical experiences
More possible side effects	
